# CD44 impacts glomerular parietal epithelial cell changes in the aged mouse kidney

**DOI:** 10.14814/phy2.14487

**Published:** 2020-06-29

**Authors:** Hiroko Hamatani, Diana G. Eng, Keiju Hiromura, Jeffrey W. Pippin, Stuart J. Shankland

**Affiliations:** ^1^ Division of Nephrology University of Washington School of Medicine Seattle WA USA; ^2^ Department of Nephrology and Rheumatology Gunma University Graduate School of Medicine Maebashi Japan

**Keywords:** Bowman's capsule, cortical, medullary, mTOR, pERK, podocyte

## Abstract

CD44 contributes to the activation of glomerular parietal epithelial cells (PECs). Although CD44 expression is higher in PECs of healthy aged mice, the biological role of CD44 in PECs in this context remains unclear. Accordingly, young (4 months) and aged (24 months) CD44^−/−^ mice were compared to age‐matched CD44^+/+^ mice, both aged in a nonstressed environment. Parietal epithelial cell densities were similar in both young and aged CD44^+/+^ and CD44^−/−^ mice. Phosphorylated ERK 1/2 (pERK) was higher in aged CD44^+/+^ mice. Vimentin and α‐SMA, markers of changes to the epithelial cell phenotype, were present in PECs in aged CD44^+/+^ mice, but absent in aged CD44^−/−^ mice in both outer cortical (OC) and juxtamedullary (JM) glomeruli. Because age‐related glomerular hypertrophy was lower in CD44^−/−^ mice, mTOR activation was assessed by phospho‐S6 ribosomal protein (pS6RP) staining. Parietal epithelial cells and glomerular tuft staining for pS6RP was lower in aged CD44^−/−^ mice compared to aged CD44^+/+^ mice. Podocyte density was higher in aged CD44^−/−^ mice in both OC and JM glomeruli. These changes were accompanied by segmental and global glomerulosclerosis in aged CD44^+/+^ mice, but absent in aged CD44^−/−^ mice. These results show that the increase in CD44 in PECs in aged kidneys contributes to several changes to the glomerulus during healthy aging in mice, and may involve ERK and mTOR activation.

## Graphical abstract

The absence of CD44 in aged null mice was accompanied by reduced glomerulosclerosis, glomerular hypertrophy, mTOR activation, and PEC activation, supporting a probable role for CD44 in kidney aging.

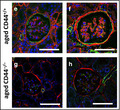

## 1 INTRODUCTION

A better understanding of kidney aging is necessary as our population is living longer, and because the severity of kidney disease increases with advancing age (Epstein, 1996; Glassock & Rule, 2012; Hommos, Glassock, & Rule, 2017; Sweetwyne et al., 2017; Wiggins, 2012). Age‐associated glomerular changes are typified by glomerular scarring and reduced podocyte density due to both a decrease in absolute podocyte number, as well as an increase in glomerular volume (Hodgin et al., 2015; Hommos et al., 2017; Kremers et al., 2015). A large body of evidence shows that a decrease in podocyte number directly correlates with both the onset and magnitude of glomerulosclerosis (Matsusaka et al., 2005; Wharram et al., 2005). Glomerular size is larger in the juxtamedullary (JM) compartment than in the outer cortical (OC) compartment (Newbold, Sandison, & Howie, 1992; Zhou et al., 2008), resulting in lower podocyte density in aged JM glomeruli compared with aged OC glomeruli (Roeder et al., 2015; Schneider et al., 2017).

A more contemporary paradigm underlying disease‐ and aged‐associated glomerulosclerosis includes a role for neighboring parietal epithelial cells (PECs) (Roeder et al., 2015; Schneider et al., 2017; Sweetwyne et al., 2017; Wiggins, Goyal, Wharram, & Wiggins, 2006; Zhang et al., 2012), in addition to podocytes. The biological function and roles of PECs are increasingly being understood in health and disease (Ohse et al., 2009; Shankland, Smeets, Pippin, & Moeller, 2014). Historically, PECs are perhaps best known for their participation in the proliferative lesion in crescentic glomerulonephritis (Smeets et al., 2009). However, following the seminal observation that PECs begin to express CD44 in certain glomerular diseases, new light has been shed on their role in glomerulosclerosis. CD44 is a cell surface glycoprotein and mediates cell‐cell and cell‐matrix interaction, proliferation, differentiation, and migration (Aruffo, Stamenkovic, Melnick, Underhill, & Seed, 1990). De novo expression of CD44 in PECs is considered to be an important marker of an “activated” state (Fatima et al., 2012; Smeets et al., 2009), defined as a migratory and profibrotic phenotype. CD44 levels increase in PECs in FSGS (Fatima et al., 2012; Kuppe et al., 2015; Smeets et al., 2011; Smeets et al., 2014), IgA nephropathy (Kim, Kim, Choi, & Jeong, 2016), and diabetic nephropathy (Holderied et al., 2015). We have reported that increased CD44 expression in PECs in experimental FSGS colocalizes with phosphorylated ERK 1/2 (pERK) (Eng et al., 2015; Roeder et al., 2017). The increase in CD44 is therefore not only a marker of PEC activation, but is also a critical mechanism underlying the PEC migratory and profibrotic phenotype in disease (Eymael et al., 2018; Roeder et al., 2017).

We have reported several changes in PECs in aged mouse kidneys, including increased CD44 expression particularly in JM glomeruli compared with OC glomeruli, increased staining for epithelial–mesenchymal transition (EMT) markers vimentin and α‐SMA, and the accumulation of the extracellular matrix proteins collagen type IV and heparin sulfate proteoglycan (Roeder et al., 2015). Several of these changes, including CD44 expression in PECs, can be limited or even prevented, by giving aged mice the mitochondrial stabilizer SS‐31 (Sweetwyne et al., 2017). The purpose of the studies described herein was to better define the role of CD44 in PECs in the healthy aged kidney, by studying CD44^−/−^ mice at an advanced age.

## 2 METHODS

### 2.1 Animals and experimental design

Breeding pairs of CD44 LacZ knockin/knockout (CD44^−/−^, B6.129(Cg)‐Cd44tm1Hbg/J, Stock #005085) (Protin, Schweighoffer, & Jochum, 1999) mice were obtained from The Jackson Laboratory (Bar Harbor, ME). CD44 wild‐type mice were obtained from the NIA aging colony (CD44^+/+^, C57BL/6). It was determined through power analysis with alpha set to 0.05, power to 0.80, that a minimum of 12 animals would be needed to meet significance based on previous data on podocyte density in aged animals. The sex distribution was as follows: young CD44^+/+^ mice (*n* = 15, all female), young CD44^−/−^ mice (*n* = 12, 7 male, 5 female), aged CD44^+/+^ mice (*n* = 15, all female), and aged CD44^−/−^ mice (*n* = 12, 8 male, 4 female).

Mice were bred, randomized, and maintained until the designated time points of 4 months (referred to as young mice, *n* = 12) or 24 months (referred to as aged mice, *n* = 12) of age in the animal care facility at the University of Washington under specific pathogen‐free conditions with ad libitum food and water, on a 14‐hr light/10‐hr dark cycle. All animals were kept in social housing with 2–5 animals per cage, with standard cage enrichment (nestlets) and were never bred. Aging animals, over 18 months of age, had an increased health monitoring protocol to ensure healthy aging. Wild‐type control (CD44^+/+^) mice at 4 months (*n* = 15) or 24 months (*n* = 15) of age were obtained from the NIH/NIA aging rodent colony and were maintained in the same room and with the same aging protocols in the animal care facility as the CD44^−/−^ animals. All CD44^−/−^ and CD44^+/+^ animals were on similar C57BL/6 backgrounds (recommended control background) and included both sexes when possible. Average weights at sacrifice for young female CD44^−/−^ and CD44^+/+^ were 25.4 ± 5.6 g and 24.4 ± 1.5 g (*p* > .05), respectively. Average weights at sacrifice for aged female CD44^−/−^ and CD44^+/+ ^mice were 28.1 ± 1.7 g and 30.2 ± 2.5 g (*p* > .05), respectively. Male CD44^−/−^ young and aged animals were 28.7 ± 1.1 g and 34.2 ± 2.2 g, respectively.

Animal protocols and procedures were designed and implemented according to the Guide for the Care and Use of Laboratory Animals, the American Veterinary Medical Association (AVMA) guidelines, and with consideration of the 3R’s of research, after review and approval by the University of Washington Institutional Animal Care and Use Committee under protocol 2968‐04. Euthanasia via cervical dislocation at designated time points were performed by certified personnel according to the AVMA guidelines for the euthanasia of animals. Mice were then perfused with ice cold PBS, the kidneys were removed, fixed in 10% neutral buffered formalin overnight, and embedded in paraffin for further analysis.

### 2.2 Immunofluorescence

For double staining of vimentin with collagen type IV or α‐SMA with collagen type IV, immunofluorescence staining was performed on 4‐µm‐thick sections as described previously (Hamatani et al., 2018). Kidney sections were deparaffinized and rehydrated, then antigen retrieval was performed by microwave heating in 10 mM citric acid buffer, pH 6.0 for α‐SMA, and pH 7.0 for vimentin. Endogenous biotin activity was blocked with an Avidin/biotin blocking kit (Vector Laboratories), and nonspecific binding was blocked with a background buster (Accurate Chemical & Scientific, Westbury, NY). Antibodies were diluted in 1% IgG‐free BSA (Sigma‐Aldrich) in PBS. Mouse anti–α‐SMA (Sigma‐Aldrich) or mouse anti‐vimentin antibody (Santa Cruz Biotechnology, Santa Cruz, CA) were incubated overnight at 4°C, followed by Alexa 488‐conjugated donkey anti‐mouse IgG (Jackson ImmunoResearch) for 1 hr at room temperature. We have reported that these antibodies colocalized with the PEC marker PAX2 (Naito, Pippin, & Shankland, 2014). Biotinylated goat anti‐collagen type IV (Southern Biotech, Birmingham, AL) was incubated overnight at 4°C, followed by Alexa 594‐conjugated streptavidin (Life Technologies) for 1 hr at room temperature. The sections were mounted with Vectashield mounting medium with DAPI (Vector Laboratories).

### 2.3 Immunohistochemistry

Immunohistochemistry for CD44, p57 with Periodic acid–Schiff (PAS) staining, PAX8, pERK, and phospho‐S6 ribosomal protein (pS6RP) was performed on 4‐µm‐thick sections. Kidney sections were deparaffinized and rehydrated, and antigen retrieval was performed by microwave heating in 10 mM citric acid buffer, pH 6.0 for CD44, pERK, and pS6RP, and 1 mM EDTA, pH 6.0 for p57 and PAX8. Endogenous peroxidase activity was blocked with 3% hydrogen peroxide. Nonspecific binding was blocked with background buster (Accurate Chemical & Scientific). Rabbit anti‐p57 (Santa Cruz Biotechnology), rabbit anti–PAX8 antibody (Protein Tech Group), rabbit anti–p‐p44/42 MAPK (pERK) (Cell Signaling Technology), or rabbit anti‐pS6RP (Cell Signaling Technology) was applied overnight at 4°C. Next ImmPRESS Reagent anti‐rabbit Ig (Vector Laboratories) was applied for 1 hr at room temperature. For CD44 staining, endogenous biotin activity was blocked with an Avidin/biotin blocking kit (Vector Laboratories) and rat anti–CD44 antibody (BD Biosciences) was applied overnight at 4°C, followed by biotinylated mouse anti–rat IgG (Jackson ImmunoResearch Laboratories) for 1 hr at room temperature, then Vectastain ABC reagent (Vector Laboratories) for 1 hr at room temperature. Color development was performed with diaminobenzidine (DAB; Thermo Fisher Scientific). Periodic acid–Schiff staining was performed after p57 staining. Counter staining was performed with hematoxylin (Sigma‐Aldrich), and the slides were dehydrated and mounted with Histomount (National Diagnostics).

### 2.4 Quantification of staining

For quantification, pictures were taken using an EVOS FL Cell Imaging System (Life Technologies) using the 20× objective. A minimum of 30 glomeruli in the OC and 15 glomeruli in the JM for each mouse were assessed. The percentage of glomeruli with pERK, vimentin, α‐SMA were assessed from each stain. For quantification of pS6RP staining in PECs, one Bowman's capsule length and total Bowman's capsule length that was covered by pS6RP staining in that glomerulus were measured using ImageJ 1.46r software (National Institutes of Health) and the percentage of Bowman's capsule covered by pS6RP^+^ PECs was calculated and averaged. For the figures, images of immunofluorescent staining were taken using a Leica TCS SPE II laser scanning confocal microscope with a 40× (1.3NA) oil objective. The percentage of segmental glomerulosclerosis and global sclerosis were assessed on p57 stained PAS counter‐stained tissue. The number of PECs was assessed by PAX8 staining, and Bowman's capsule length was measured using ImageJ. Parietal epithelial cells density was calculated as follows: the number of PAX8^+^ cells in one glomerulus was divided by the measured Bowman's capsule length in that glomerulus. The calculated PEC densities were then averaged. The number of podocytes was assessed by p57 staining and glomerular tuft area was measured using ImageJ. Podocyte density was calculated as follows: the number of p57^+^ cells in one glomerulus was divided by the measured glomerular tuft area. The calculated podocyte densities were then averaged. For quantification of pS6RP staining in the glomerular tuft area, the glomerular tuft area and the pS6RP stained area in the glomerular tuft were measured using ImageJ and the percentage of the glomerular tuft area with pS6RP was calculated and averaged.

### 2.5 Statistical analyses

Data are expressed as means ± *SD*. The one‐way ANOVA with Tukey's multiple‐comparisons test was performed using GraphPad Prism (version 7.0; GraphPad Software). A *p*‐value <.05 was considered significant.

## 3 RESULTS

### 3.1 PEC activation defined by pERK staining was lower in aged CD44^−/−^ mice

Parietal epithelial cell activation is defined as the de novo expression of CD44 (Fatima et al., 2012; Smeets et al., 2009) and pERK (Eng et al., 2015; Roeder et al., 2017). Young and aged animals in both the CD44^−/−^ and CD44^+/+^ cohorts were phenotypically healthy at the time of sacrifice, in line with expectations for healthy aging, with no obvious external deficits in any group as a result of the presence or absence of CD44. Upon histological analysis of kidneys, CD44 staining was expressed in PECs in aged CD44^+/+^ mice as reported (Roeder et al., 2015), but was absent in aged CD44^−/−^ mice as expected, as we have previously reported (Roeder et al., 2017) (data not shown).

**FIGURE 1:**
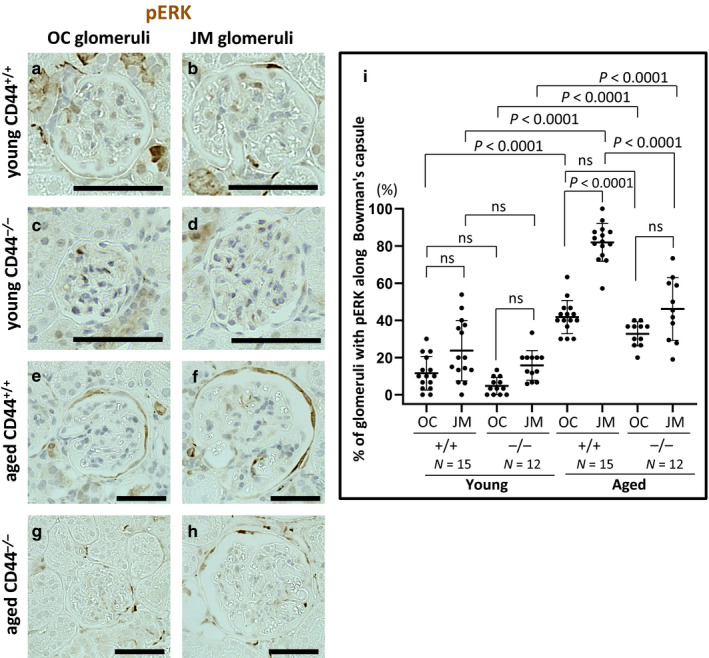
pERK in PECs was lower in aged CD44^−/−^ mice. (a–h) Immunoperoxidase staining for pERK (brown color) in outer cortical (OC) and juxtamedullary (JM) glomeruli in young (a–d) and aged (e–h) CD44^+/+^ and CD44^−/−^ mice. (i) Quantification of the percentage of glomeruli with pERK staining in PECs along Bowman's capsule was higher in OC and JM glomeruli in aged CD44^+/+^ and CD44^−/−^ mice compared to young mice. In aged CD44^+/+^ mice, pERK staining was higher in JM glomeruli than in OC glomeruli, with no difference between OC and JM glomeruli in aged CD44^−/−^ mice. Scale bars represent 50 µm. NS indicates not significant

Representative examples of pERK staining are shown in Figure 1a–h, and the quantitation is shown in Figure 1i. In aged CD44^+/+^ mice compared to young CD44^+/+^ mice, pERK increased 3.6‐fold in PECs OC glomeruli (41.78 ± 8.90% vs 11.56 ± 9.07%, *p* < .0001 vs. young), and increased 3.5‐fold in JM glomeruli (81.95 ± 10.15% vs. 23.73 ± 16.24%, *p* < .0001 vs. young). Overall, JM glomeruli had higher pERK staining along Bowman's capsule compared to OC glomeruli in aged CD44^+/+^ mice (81.95 ± 10.15% vs. 41.78 ± 8.90%, *p* < .0001 vs. OC).

Compared to young CD44^−/−^ mice, pERK staining in PECs in aged CD44^−/−^ mice increased 6.9‐fold in OC glomeruli (32.73 ± 6.47% vs. 4.72 ± 4.60%, *p* < .0001 vs. young) and 2.9‐fold in JM glomeruli (46.10 ± 16.81% vs. 15.78 ± 7.97%, *p* < .0001 vs. young) (Figure 1i). Overall there were no differences in pERK staining between OC and JM glomeruli in aged CD44^−/−^ mice (46.10 ± 16.81% vs. 32.73 ± 6.47%, *p* = .086 vs. OC).

Compared with aged CD44^+/+^ mice, pERK staining was lower in PECs in JM glomeruli of aged CD44^−/−^ mice (*p* < .0001), with no differences in pERK staining in OC glomeruli (*p* = .42). These results show that pERK increased in PECs in both aged CD44^+/+^ and CD44^−/−^ mice but was lower in aged CD44^−/−^ mice, compared to aged CD44^+/+^ mice.

### 3.2 Changes to the PEC phenotype was lower in aged CD44^−/−^ mice

**FIGURE 2:**
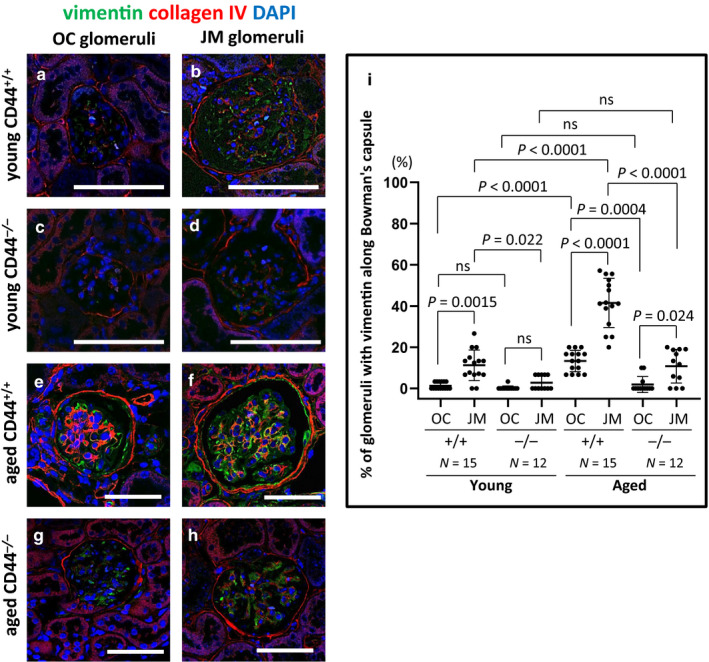
Vimentin in PECs was higher in aged CD44^+/+^ mice than in aged CD44^−/−^ mice. (a–h) Representative examples of vimentin (green) and collagen IV (red) co‐immunofluorescent staining in outer cortical (OC) and juxtamedullary (JM) glomeruli. Collagen IV was used to readily identify Bowman's capsule. DAPI (blue) identified nuclei. (i) Quantitation of the percentage of glomeruli vimentin staining along Bowman's capsule. The percentage of OC and JM glomeruli with vimentin staining in PECs was higher in aged CD44^+/+^ mice compared to young mice, but not in aged CD44^−/−^ mice. Scale bars represent 50 µm. NS indicates not significant

**FIGURE 3:**
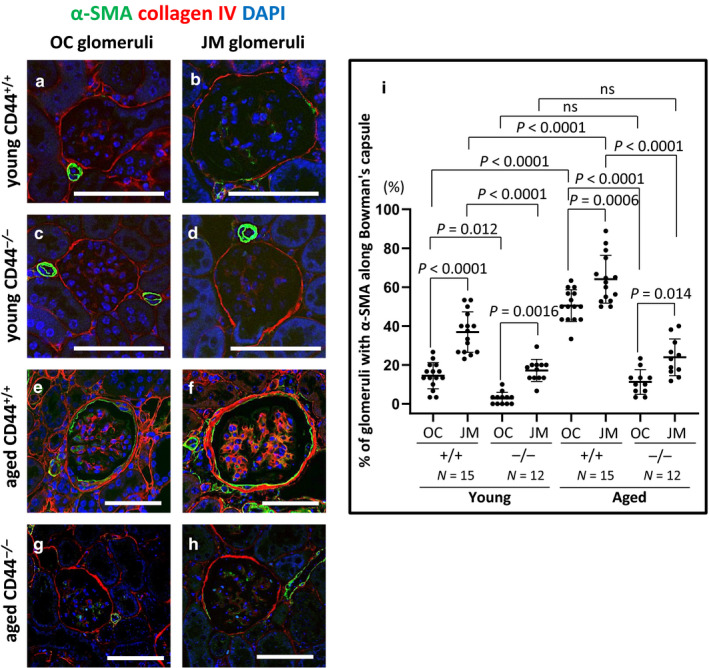
α‐SMA staining in PECs was higher in aged CD44^+/+^ mice than in aged CD44^−/−^ mice. (a–h) Representative examples of α‐SMA (green) and collagen IV (red) co‐immunofluorescent staining in outer cortical (OC) and juxtamedullary (JM) glomeruli. Collagen IV was used to readily identify Bowman's capsule. DAPI (blue) identified nuclei. (i) Quantitation of the percentage of glomeruli with α‐SMA staining along Bowman's capsule. The percentage of OC and JM glomeruli with α‐SMA staining in PECs was higher in aged CD44^+/+^ mice compared to young mice, but not in aged CD44^−/−^ mice. α‐SMA staining in PECs was higher in JM glomeruli than in OC glomeruli in both aged CD44^+/+^ and CD44^−/−^ mice. Scale bars represent 50 µm. NS indicates not significant

Vimentin (Figure 2) and α‐SMA (Figure 3) were used as markers of changes from the typical PEC epithelial phenotype. Representative examples of staining for vimentin are shown in Figure 2a–h. Double staining was performed with collagen IV to readily identify Bowman's capsule. Quantification of the percentage of glomeruli with vimentin staining along Bowman's capsule is shown in Figure 2i. In aged CD44^+/+^ mice, the percentage of glomeruli with vimentin staining along Bowman's capsule increased 10‐fold among OC glomeruli (13.33 ± 5.19% vs. 1.33 ± 1.69, *p* < .0001 vs. young CD44^+/+^ mice) and 3.7‐fold among JM glomeruli (41.51 ± 11.94% vs. 11.27 ± 7.39%, *p* < .0001 vs. young CD44^+/+^ mice) (Figure 2i). Overall, the percentage of glomeruli with PEC vimentin staining was significantly higher in JM glomeruli than in OC glomeruli in aged CD44^+/+^ mice (41.51 ± 11.94% vs. 13.33 ± 5.19%, *p* < .0001 vs. OC).

In aged CD44^−/−^ mice, the percentage of glomeruli with vimentin staining along Bowman's capsule did not increase in either OC glomeruli (1.94 ± 3.88% vs. 0.28 ± 0.96%, *p* > .05 vs. young) or JM glomeruli (10.81 ± 8.15% vs. 2.78 ± 3.43%, *p* = .059 vs. young) (Figure 2i). Overall in aged CD44^−/−^ mice, the percentage of glomeruli with vimentin staining in PECs was higher in JM glomeruli (10.81%±8.15% vs. 1.94%±3.88%, *p* = .024 vs. OC).

Compared with aged CD44^+/+^ mice, aged CD44^−/−^ mice had significantly less glomeruli with PEC staining for vimentin in both OC and JM glomeruli (*p* = .0004 and *p* < .0001, respectively). These results show that vimentin staining in PECs along Bowman's capsule was lower in healthy aged CD44^−/−^ mice compared to similarly healthy aged CD44^+/+^ mice.

Representative examples of staining for α‐SMA are shown in Figure 3a–h. Double staining was performed with collagen IV to readily identify Bowman's capsule. Quantification of the percentage of glomeruli with α‐SMA staining along Bowman's capsule is shown in Figure 3i. Compared to young CD44^+/+^ mice, the percentage of glomeruli with α‐SMA staining in PECs along Bowman's capsule in aged CD44^+/+^ mice was 3.5‐fold higher among OC glomeruli (50.44 ± 8.15% vs. 14.44 ± 6.75%, *p* < .0001 vs. young) and 1.7‐fold among JM glomeruli (64.08 ± 12.31% vs. 36.87 ± 10.41%, *p* < .0001 vs. young). Compared to young CD44^−/−^ mice, α‐SMA staining in PECs along Bowman's capsule in aged CD44^−/−^ mice did not increase in either OC glomeruli (11.21 ± 6.37% vs. 2.78 ± 3.12%, *p* > .05 vs. young) or JM glomeruli (23.90 ± 9.49% vs. 17.15 ± 5.67%, *p* > .05 vs. young). Compared with aged CD44^+/+^ mice, aged CD44^−/−^ mice had significantly lower α‐SMA staining in PECs along Bowman's capsule in both OC and JM glomeruli (*p* < .0001, *p* < .0001, respectively).

These results show that age‐associated changes in the PEC phenotype were lower in CD44^−/−^ mice compared to similar aged CD44^+/+^ mice.

### 3.3 Glomerulosclerosis was lower in aged CD44^−/−^ mice

#### 3.3.1 Segmental sclerosis

**FIGURE 4:**
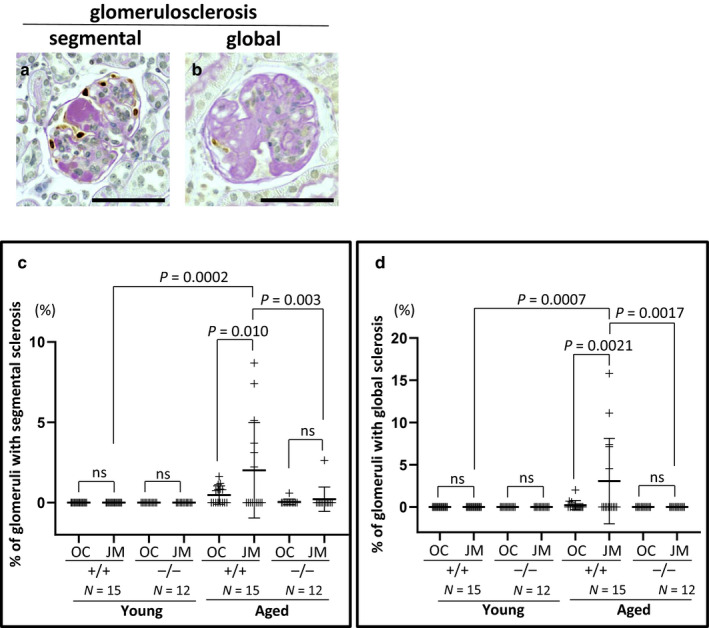
Segmental and global glomerulosclerosis were lower in aged CD44^−/−^ mice. (a and b) Representative images of the criteria used to define segmental (a) and global (b) glomerulosclerosis. (c) Segmental glomerulosclerosis was higher in JM glomeruli of aged CD44^+/+^ mice compared to their young counterparts and to aged CD44^−/−^ mice. Segmental glomerulosclerosis did not increase in aged CD44^−/−^ mice. (d) Global glomerulosclerosis increased in JM glomeruli of aged CD44^+/+^ mice, but was not detected in either the OC or JM glomeruli of aged CD44^−/−^ mice. Scale bars represent 50 µm. NS indicates not significant

Compared with young CD44^+/+^ mice, aged CD44^+/+^ mice had a significant increase in the percentage of glomeruli with segmental sclerosis (example shown in Figure 4a) in JM glomeruli (*p* = .0002), but not in OC glomeruli (Figure 4c). In aged CD44^+/+^ mice, the percentage of glomeruli with segmental sclerosis was higher in JM glomeruli when compared to OC glomeruli (2.02 ± 2.98% vs. 0.48 ± 0.57%, *p* = .010 vs. OC). However, in CD44^−/−^ mice, there was no difference in the percentage of glomeruli with segmental sclerosis between young and aged mice in either OC or JM glomeruli, nor was there a difference in the percentage of glomeruli with segmental sclerosis between OC and JM glomeruli in aged CD44^−/−^ mice (0.22 ± 0.76% vs. 0.049 ± 0.17%, *p* > .05 vs. OC).

There were no statistical differences in the percentage of glomeruli with segmental sclerosis in OC in aged CD44^+/+^ compared to aged CD44^−/−^ mice. In contrast, the percentage of glomeruli with segmental sclerosis was significantly lower in JM glomeruli in aged CD44^−/−^ compared with aged CD44^+/+^ mice (*p* = .003).

#### 3.3.2 Global sclerosis

In aged CD44^+/+^ mice, the percentage of glomeruli with global sclerosis (example shown in Figure 4b) was higher in the JM glomeruli compared to OC glomeruli (3.07 ± 5.06% vs. 0.22 ± 0.54%, *p* = .0021 vs. OC) (Figure 4d). In contrast, global sclerosis was not observed in either OC or JM glomeruli in aged CD44^−/−^ mice.

These results are consistent with aged CD44^−/−^ mice developing less segmental and global sclerosis in both OC and JM glomeruli, whereas aged CD44^+/+^ mice had an increase in segmental and global glomerulosclerosis in JM glomeruli only.

### 3.4 CD44 levels did not impact PEC density in aged mice

**FIGURE 5:**
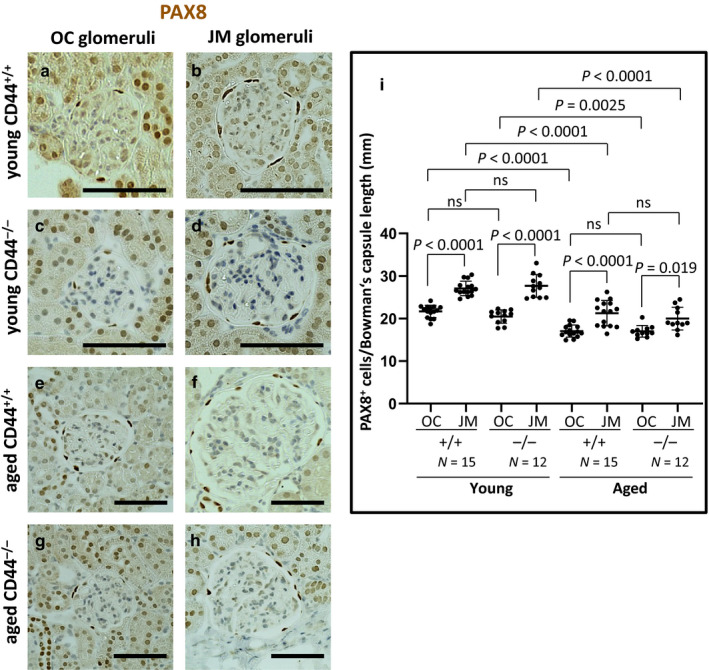
PEC density was similar in aged CD44^+/+^ and CD44^−/−^ mice. (a–h) Immunoperoxidase staining for PAX8 was used to measure PEC number in outer cortical (OC) and juxtamedullary (JM) glomeruli. (i) PEC density was quantitated by measuring the number of PAX8 stained cells along Bowman's capsule, and dividing that by the length of Bowman's capsule in individual glomeruli. PEC density did not differ between aged CD44^+/+^ and aged CD44^−/−^ mice. Scale bars represent 50 µm. NS indicates not significant

Representative examples of PAX8 staining, a PEC marker, are shown in Figure 5a–h. PEC density, defined as the number of PAX8 stained cells/Bowman's capsule length (mm) was measured and the results are shown in Figure 5i. Three results emerged: (a) In young and aged CD44^+/+^ and CD44^−/−^ mice, PEC density was significantly higher in JM glomeruli compared with OC glomeruli as follows: young CD44^+/+^ mice (27.08 ± 1.70 cells/mm vs. 21.67 ± 1.44 cells/mm, *p* < .0001 vs. OC), young CD44^−/−^ mice (27.68 ± 2.66 cells/mm vs. 20.47 ± 1.56 cells/mm, *p* < .0001 vs. OC), aged CD44^+/+^ mice (21.26 ± 2.99 cells/mm vs. 17.05 ± 1.41 cells/mm, *p* < .0001 vs. OC), and aged CD44^−/−^ mice (19.99 ± 2.67 cells/mm vs. 16.98 ± 1.36 cells/mm, *p* = .019 vs. OC). (b) Compared to young mice, PEC density was lower in both OC and JM glomeruli in both strains of aged mice. (c) There were no differences in PEC density between aged CD44^+/+^ and CD44^−/−^ mice in either the OC or JM glomeruli. These results show that age‐related changes due to the absence of CD44 are unlikely due to differences in PEC density, which were similar between CD44^+/+^ and CD44^−/−^ mice.

### 3.5 Podocyte density was higher in aged CD44^−/−^ mice

**FIGURE 6:**
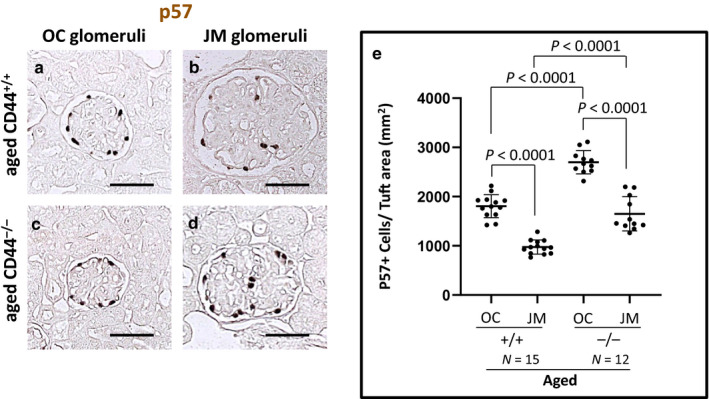
Podocyte density was lower in aged CD44^−/−^ mice. (a–d) Representative immunoperoxidase staining for p57, used to measure podocyte number in outer cortical (OC) and juxtamedullary (JM) glomeruli. (e) Podocyte density was quantitated by measuring the number of p57 stained nuclei, divided by the glomerular tuft area. Podocyte density was lower in both OC and JM glomeruli of aged CD44^+/+^ mice compared to aged CD44^−/−^ mice. Scale bars represent 50 µm. NS indicates not significant

Podocyte density (number/area) was calculated by dividing the number of p57 stained nuclei by the glomerular tuft area (mm^2^). We have reported that podocyte density is similar in young CD44^−/−^ and young CD44^+/+^ mice (Roeder et al., 2017). Representative examples of p57 staining are shown in Figure 6a–d, and quantitation is shown in Figure 6e. Podocyte density was lower in JM glomeruli than in OC glomeruli in both CD44^+/+^ and CD44^−/−^ mice as follows: aged CD44^+/+^ (975 ± 144 cells/mm^2^ vs. 1805 ± 235 cells/mm^2^, *p* < .0001 vs. OC), aged CD44^−/−^ (1649 ± 348 cells/mm^2^ vs. 2,699 ± 236 cells/mm^2^, *p* < .0001 vs. OC). Podocyte density was higher in aged CD44^−/−^ versus CD44^+/+^ mice in both OC and JM glomeruli (*p* < .0001, *p* < .0001, respectively). These results show that in the absence of CD44, podocyte density was maintained in aged mice.

### 3.6 Glomerular size differed between CD44^+/+^ and CD44^−/−^ mice

**FIGURE 7:**
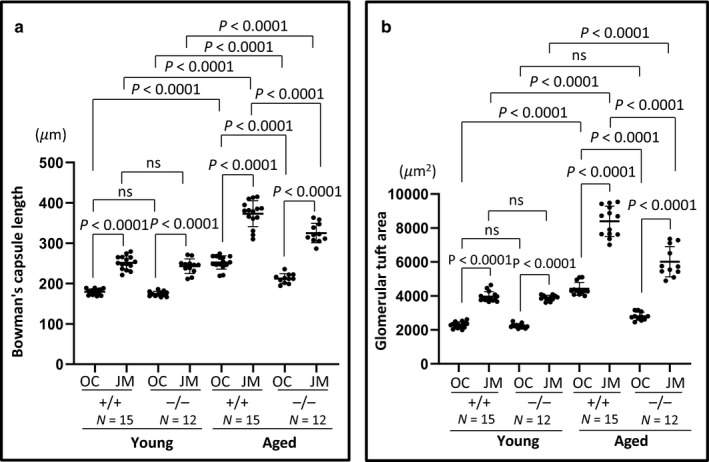
Glomerular size was smaller in aged CD44^−/−^ mice. (a) Bowman's capsule length was similar between young CD44^+/+^ and CD44^−/−^ mice. In aged mice, Bowman's capsule length was higher in CD44^+/+^ mice than in CD44^−/−^ mice. (b) Glomerular tuft area was similar between young CD44^+/+^ and CD44^−/−^ mice. In aged mice, glomerular tuft area was higher in CD44^+/+^ mice than in CD44^−/−^ mice. NS indicates not significant

There were no differences in Bowman's capsule length between young CD44^+/+^ and CD44^−/−^ mice in both JM and OC glomeruli. As expected, JM glomeruli had longer Bowman's capsule length compared to OC glomeruli (Figure 7a). Compared to young CD44^+/+^ mice, Bowman's capsule length in aged CD44^+/+^ increased 1.4‐fold and 1.5‐fold in OC and JM glomeruli, respectively. Compared to young CD44^−/−^ mice, Bowman's capsule length in aged CD44^−/−^ increased 1.2‐fold and 1.3‐fold in OC and JM glomeruli, respectively. However, Bowman's capsule length was lower in aged CD44^−/−^ mice compared to aged CD44^+/+^ mice in both OC (212.42 ± 11.74 µm vs. 252.36 ± 16.50 µm, *p* < .0001 vs. CD44^+/+^) and JM glomeruli (325.06 ± 24.02 µm vs. 373.05 ± 32.16 µm, *p* < .0001 vs. CD44^+/+^).

The glomerular tuft area was similar in young CD44^+/+^ and CD44^−/−^ mice in both OC and JM glomeruli (Figure 7b), and increased with aging in both. However, the glomerular tuft area was significantly lower in aged CD44^−/−^ mice compared with aged CD44^+/+^ mice in both OC (2,801 ± 241 µm^2^ vs. 4,415 ± 379 µm^2^, *p* < .0001 vs. CD44^+/+^) and JM glomeruli (6,011 ± 823 µm^2^ vs. 8,392 ± 892 µm^2^, *p* < .0001 vs. CD44^+/+^). With aging, JM glomeruli remain larger than OC glomeruli in both aged CD44^+/+^ and aged CD44^−/−^ mice (*p* < .0001, *p* < .0001, respectively) (Figure 7b). All animals analyzed were noted as healthy, with similar average body weights in aged CD44^+/+^ and CD44^−/−^ mice, suggesting that obesity/body size or other apparent physical comorbidities did not impact the results.

Taken together, both Bowman's capsule length and glomerular tuft area increased with age but were lower in aged CD44^−/−^ mice, compared to aged CD44^+/+^ mice.

### 3.7 pS6RP staining in PECs and on the glomerular tuft was lower in aged CD44^−/−^ mice

**FIGURE 8:**
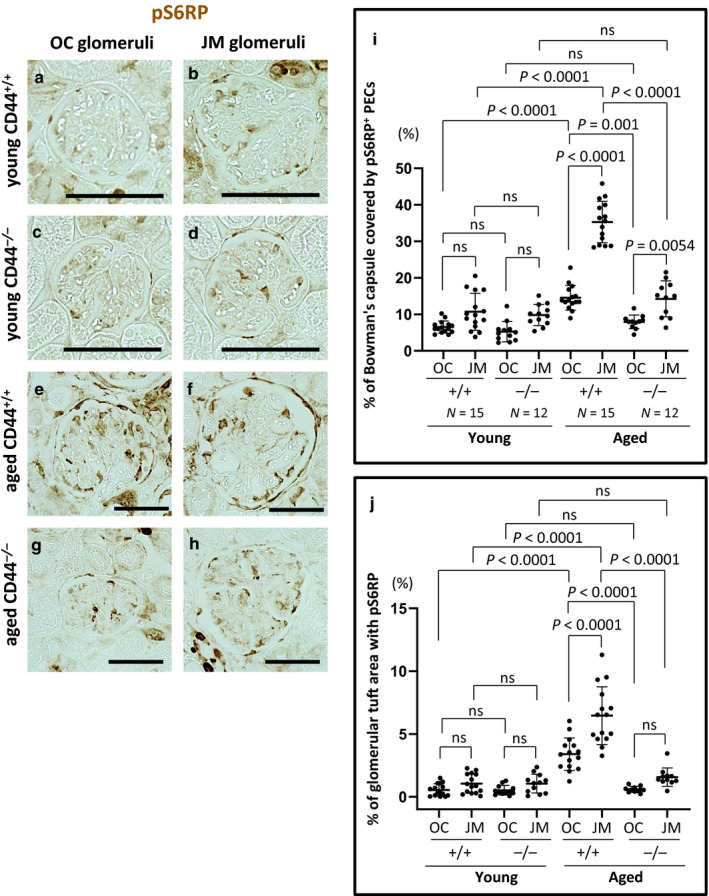
mTOR activity was higher in aged CD44^+/+^ mice. (a–h) Representative examples of immunoperoxidase staining for pS6RP. (i) Quantitation of pS6RP staining along Bowman's capsule. The percentage of Bowman's capsule covered by pS6RP^+^ PECs was higher in aged CD44^+/+^ mice than in aged CD44^−/−^ mice. (j) Quantitation of pS6RP staining on the glomerular tuft. The percentage of the glomerular tuft area with pS6RP staining was higher in aged CD44^+/+^ mice than in aged CD44^−/−^ mice. Scale bars represent 50 µm. NS indicates not significant

We (Hamatani et al., 2014; McNicholas et al., 2016) and others (Kurayama et al., 2011) showed important roles for the mTOR pathway in PECs in aging and disease, respectively. Therefore, pS6RP staining was used herein as a measure of mTOR activity (Hamatani et al., 2014; McNicholas et al., 2016). Representative examples of pS6RP staining are shown in Figure 8a–h. Quantification of the percentage of Bowman's capsule length that was covered by pS6RP staining was measured and the results are shown in Figure 8i. In aged CD44^+/+^ mice compared to young CD44^+/+^ mice, pS6RP staining in PECs increased 2.2‐fold in OC glomeruli (14.54 ± 3.39% vs. 6.50 ± 1.66%, *p* < .0001 vs. young) and 3.3‐fold in JM glomeruli (35.28 ± 5.68% vs. 10.69 ± 5.09%, *p* < .0001 vs. young). Within aged CD44^+/+^ mice, pS6RP staining in PECs was significantly higher in JM glomeruli (35.28 ± 5.68% vs. 14.54 ± 3.39%, *p* < .0001 vs. OC).

There were no differences in the percentage of Bowman's capsule covered by pS6RP stained PECs between aged and young CD44^−/−^ mice in either OC (7.93 ± 1.86% vs. 5.24 ± 2.80%, *p* > .05 vs. young) or JM glomeruli (14.24 ± 4.92% vs. 9.82 ± 2.88%, *p* > .05 vs. young). Compared with aged CD44^+/+^ mice, pS6RP stained PECs were significantly lower in aged CD44^−/−^ mice in both OC and JM glomeruli (*p* = .001, *p* < .0001, respectively).

These results show that an age‐associated increase in mTOR activation in PECs was significantly less in aged CD44^−/−^ mice when compared with aged CD44^+/+^ mice.

Because glomerular hypertrophy has been associated with mTOR activation in diabetes (Nagai et al., 2005), we also measured mTOR activation in the glomerular tuft area by quantifying pS6RP staining (Figure 8j). In aged CD44^+/+^ mice compared to young CD44^+/+^ mice, pS6RP staining in the glomerular tuft area increased 6.3‐fold in OC glomeruli (3.40 ± 1.29% vs. 0.54 ± 0.49%, *p* < .0001 vs. young) and 6.2‐fold in JM glomeruli (6.46 ± 2.30% vs. 1.05 ± 0.77%, *p* < .0001 vs. young). Within aged CD44^+/+^ mice, pS6RP staining in glomerular tuft area was significantly higher in JM glomeruli (*p* < .0001 vs. OC). There were no differences in pS6RP staining in the glomerular tuft area between aged and young CD44^−/−^ mice in either OC (0.59 ± 0.23% vs. 0.52 ± 0.39%, *p* > .05 vs. young) or JM glomeruli (1.56 ± 0.74% vs. 1.05 ± 0.75%, *p* > .05 vs. young). Compared with aged CD44^+/+^ mice, pS6RP staining was significantly lower in aged CD44^−/−^ mice in both OC and JM glomeruli (*p* < .0001, *p* < .0001, respectively) (Figure 8j).

These results show that age‐associated increase in mTOR activation in glomerular tuft area was lower in aged CD44^−/−^ mice compared with aged CD44^+/+^ mice.

## 4 DISCUSSION

The histopathological and molecular changes in the healthy aged kidney are being increasingly recognized in order to better understand their impact on the severity and prognosis when kidney diseases are superimposed on an increasing elderly population. The current thinking around glomerulosclerosis, a hallmark of kidney aging, is evolving from the original podocyte‐centric view, to one that also includes the activated glomerular parietal epithelial cell (PEC), often defined as de novo expression of CD44 (Fatima et al., 2012; Smeets et al., 2009), and increased pERK (Eng et al., 2015; Roeder et al., 2017). The results of the current study show that when CD44 is absent in aged CD44^−/−^ mice (equivalent to human aged 70 years) (Fox et al., 2006), PEC activation (pERK staining), PEC phenotypic changes (increased vimentin and α‐SMA expression), PEC mTOR activation (pS6RP staining), and glomerular size were lower than in age‐matched wild‐type mice, and that these events were accompanied by reduced global and segmental glomerulosclerosis. Podocyte density was higher in aged CD44^−/−^ mice, whereas PEC densities were similar in CD44^+/+^ and CD44^−/−^ mice.

pERK increased in PECs in both aged CD44^+/+^ and CD44^−/−^ mice, consistent with PEC activation (Roeder et al., 2015). Interestingly however, was that pERK staining was lower in JM glomeruli of aged CD44^−/−^ mice compared to aged CD44^+/+^ mice. The absence of CD44 and lower levels of pERK are consistent with lower PEC activation in JM glomeruli in aged CD44^−/−^ mice, and likely OC glomeruli too, compared to aged CD44^+/+^ mice. This leads to question why pERK might be lower in JM glomeruli of aged CD44^−/−^ mice, because we have reported that pERK is an upstream regulator of CD44 in cultured PECs (Roeder et al., 2017), and therefore one would not expect any changes in pERK in the absence of CD44. In the absence of U0126 inhibitor studies in cultured CD44^+/+^ and CD44^−/−^ parietal epithelial cells from JM glomeruli, this cannot be delineated. The reverse is also true that CD44 can regulate pERK in cancer cells (Bourguignon, Gilad, Rothman, & Peyrollier, 2005; Herishanu et al., 2011; Yu et al., 2015), suggesting that the pERK—CD44 pathway in PECs may be context‐ and cell‐ dependent, and even raises the possibility of differences in regulation between OC and JM glomeruli.

PECs undergo phenotypic changes in aging, including increased expression of α‐SMA and vimentin (Roeder et al., 2015). Some consider these as consistent with a switch from an epithelial phenotype to a mesenchymal one. Both α‐SMA and vimentin increased in aged wild‐type mice. In contrast, neither α‐SMA nor vimentin increased in OC and JM glomeruli of aged CD44^−/−^ mice compared to their younger counterparts. Taken together, two markers of changes to the epithelial phenotype (α‐SMA and vimentin) increase in PECs of healthy aged CD44^+/+^ mice, but not in healthy aged CD44^−/−^ mice. We therefore speculate that the lower levels of α‐SMA and vimentin staining in aged CD44^−/−^ PECs reflect an absence of CD44, suggesting a causal role for CD44. The mechanisms underlying this need further study.

Similar to our previous report (Roeder et al., 2015), segmental and global glomerulosclerosis are higher in the JM glomeruli of aged wild‐type mice compared to OC glomeruli. In the current study, we focused on CD44 in aged PECs, because we (Roeder et al., 2015) have shown that CD44 increases in PECs in aged kidneys, and we (Roeder et al., 2017) and others (Fatima et al., 2012; Smeets et al., 2009) have shown that increased CD44 is a major cause for PEC activation, typified as a promigratory and profibrotic phenotype. Neither segmental nor global glomerulosclerosis increased in aged CD44^−/−^ mice. Glomerular lesions are typically lower in aged female mice than age‐matched male mice. In this study, however, the female aged CD44^+/+^ mice had more glomerulosclerosis than the male CD44^−/−^ mice. These interesting results suggest that the determinant of outcomes for scarring is the presence or absence of the CD44 gene, and that sex as a biological variable was less important in this study. We can therefore add healthy aging to the kidney studies performed in CD44^−/−^ mice that attenuates histological damages in crescentic glomerulonephritis and FSGS (Eymael et al., 2018), obstructive nephropathy (Rouschop et al., 2004), renal ischemia reperfusion injury (Rouschop et al., 2005), and lipopolysaccharide (LPS)‐induced acute kidney injury (Rampanelli et al., 2013). We cannot exclude the impact of changes to the PEC phenotype, described earlier, as an explanation for differences in glomerulosclerosis. However, the maintenance of a higher density of podocytes in CD44^−/−^ mice may be responsible.

The serine–threonine protein kinase mammalian target of rapamycin (mTOR) regulates cell growth, proliferation, protein synthesis, transcription, and autophagy. mTOR activation underlies glomerular hypertrophy in diabetic nephropathy (Nagai et al., 2005) and chronic kidney disease (Lieberthal & Levine, 2009), and mTOR inhibition prevents diabetic glomerular hypertrophy (Inoki et al., 2011). pS6RP expression, a downstream target of mTOR, increases in PECs in FSGS and healthy aging in mice (McNicholas et al., 2016), in experimental injury models including adriamycin nephropathy, puromycin aminonucleoside nephropathy, and crescentic glomerulonephritis in the rat (Hamatani et al., 2014; Kurayama et al., 2011). In the current study, we confirmed that pS6RP expression is increased in PECs in aged CD44^+/+^ mice, similar to what we have previously reported in another mouse strain (McNicholas et al., 2016). An unexpected finding in the current study was that pS6RP expression was significantly lower in PECs and on the glomerular tuft of aged CD44^−/−^ mice (in both OC and JM glomeruli) compared to aged CD44^+/+^ mice. Indeed, studies in non–kidney cells shows a correlation between CD44 and mTOR, and that lowering CD44 results in lower mTOR signaling (Daley et al., 2013; Gadhoum, Madhoun, Abuelela, & Merzaban, 2016; Li et al., 2018). This begs the question of what the potential biological significance is of a lower active mTOR pathway in the absence of CD44? Contrary to our report that mTORC1 inhibition by rapamycin in aged mice increased PEC density (McNicholas et al., 2016), PEC density was unchanged in aged CD44^−/−^ mice with lower mTOR activity. However, lower mTOR correlated in this study with reduced glomerular size, as both Bowman's capsule length and glomerular tuft size were lower in aged CD44^−/−^ mice compared to aged CD44^+/+^ mice with higher active mTOR in PECs. Further studies with inhibition of mTORC1 in cultured CD44^+/+^ and CD44^−/−^ parietal epithelial cells, are needed to delineate the biological consequences. Taken together, one might speculate that the absence of any increase in CD44 in aged mice limits glomerulosclerosis through several mechanisms: reduced PEC activation, reduced changes from the epithelial phenotype, and reduced glomerular hypertrophy.

We recognize several limitations in this study. First, we did not measure kidney weight, although glomerular size is not associated with kidney size in some studies (MacKay, Striker, Stauffer, Agodoa, & Striker, 1990). Second, the molecular mechanisms underlying the changes in PECs in the absence of CD44 are not delineated. Third, we used a global CD44 knockout/knockin mouse, rather than a tissue specific deletion. This might be relevant because CD44 is expressed on other cells, as well PECs, such as circulating cells, which might impact outcomes in the absence of CD44. This is less likely because there are very little if any infiltrating cells in aged mouse glomeruli. Fourth, aging in mice under controlled laboratory conditions are different to human aging. Finally, the mechanisms of glomerular scarring are likely multi‐factorial, that might include the changes to PECs being secondary to other factors such as reduced podocyte number and increased single nephron glomerular filtration rate (Denic et al., 2017), and cross talk from podocytes to PECs as recently described by Ito, Sakamoto, Hikichi, Matsusaka, & Nagata (2020).

In summary, we demonstrated that in CD44^−/−^ mice, the lack of functional CD44, limited age‐related increases in segmental and global glomerulosclerosis, glomerular hypertrophy, an increase in Bowman's capsule length, changes in the PEC phenotype, PEC ERK activation, and mTOR activation. Therefore, CD44 is likely associated with these changes.

## CONFLICT OF INTEREST

No conflicts of interest, financial, or otherwise, are declared by the authors.

## AUTHOR CONTRIBUTIONS

H.H. and D.G.E. performed experiments; H.H., J.W.P., and S.J.S. analyzed data; H.H., J.W.P., and S.J.S. interpreted results of experiments; H.H. prepared figures; H.H., D.G.E., K.H., J.W.P., and S.J.S. drafted manuscript; D.G.E., J.W.P., and S.J.S. conceived and designed research; D.G.E., J.W.P., and S.J.S. edited and revised manuscript; S.J.S. approved final version of manuscript.

## ETHICAL STATEMENT

All animal experiments were reviewed approved by the University of Washington Institutional Animal Care and Use Committee (IACUC) protocol 2968‐04. The results presented in this paper have not been published previously, in whole or in part.
